# Electromyography in abdominal laparoscopic and robot-assisted laparoscopic surgery: A scoping review

**DOI:** 10.1371/journal.pone.0354158

**Published:** 2026-08-03

**Authors:** Matthew Davitt, Christopher J. Gaffney, Daren Subar, Lawrence D. Hayes

**Affiliations:** 1 Lancaster Medical School, Lancaster University, Lancaster, United Kingdom; 2 East Lancashire Teaching Hospitals Trust NHS, Lancaster, United Kingdom; Jonkoping University, SWEDEN

## Abstract

**Purpose:**

Technological advancements have expanded options available for minimally invasive surgery, particularly laparoscopic surgery (LS) and robot-assisted laparoscopic surgery (RALS). However, these developments raise important questions about their impact on surgeon performance and well-being. This scoping review aimed to systematically map the current literature on the use of electromyography (EMG) to assess muscle activation in surgeons performing LS and RALS.

**Methods:**

This study was a scoping review of the literature and did not require ethical approval. This review was conducted following the Joanna Briggs Institute (JBI) guidelines and the PRISMA-ScR checklist. A comprehensive search was performed across multiple databases including MEDLINE, Scopus, PubMed, EMBASE, and others. Studies were screened and selected using the Population, Concept, and Context (PCC) framework. Data were charted using a custom extraction form and analysed descriptively. Studies were included if they took place in a surgical setting (including simulated environments) involving laparoscopic abdominal surgery and used EMG to measure electrical activity, ergonomics, or muscular stress of a muscle group.

**Results:**

Ninety-four unique studies published between 1997 and 2025 were included. Most studies focussed on LS (n = 62), with fewer investigating RALS (n = 5), comparing LS and RALS directly (n = 12) or isolated laparoscopic task settings (n = 15). EMG was primarily used to assess muscle activation in the deltoids, trapezius, and forearm muscles. Studies employed various methodologies, including root mean squared (RMS) amplitude and median frequency analysis, and often integrated EMG with other tools such as EEG, kinematic analysis, and questionnaires. Within the subset of comparative studies between RALS and LS, findings consistently suggested lower muscle activation in RALS compared to LS, particularly in the upper body musculature. However, this should be interpreted cautiously because the comparative evidence base is relatively small and heterogeneous.

**Conclusions:**

EMG is a well-established and versatile tool for evaluating the ergonomic demands of minimally invasive surgery. While the broader literature maps the muscular demands of LS, a specific trend within comparative studies suggests RALS may reduce electrical activity compared to LS, especially in the shoulders and upper back. However, methodological variability and limited longitudinal data highlight the need for standardised protocols and further research. Future studies should explore long-term musculoskeletal outcomes and integrate EMG into surgical training to enhance ergonomic awareness. A future meta-analysis may be feasible, but only for carefully defined subgroups with comparable EMG protocols, task types, muscle groups, normalization procedures, and outcome measures.

## Introduction

Abdominal minimally invasive surgery (MIS), including both laparoscopic surgery (LS) and robot-assisted laparoscopic surgery (RALS), has transformed the surgical field, allowing for minimally invasive alternatives to the traditional use of open surgical techniques. Research has suggested that LS often reduces post-operative hospital stays, recovery times, improves cosmetic outcomes, and shortens surgery length compared to traditional surgery, although reports vary between populations and procedures [1]. However, LS introduces ergonomic challenges, including prolonged improper static postures and constrained instrument manipulation, which contribute to significant musculoskeletal strain in surgeons [2]. This causes both acute and chronic musculoskeletal disorders and fatigue, leading to reduced length of surgeon careers, increase work absence, general reductions in well-being, and potential reductions in quality of patient care.

The introduction of RALS has offered a new paradigm for minimally invasive surgery, with these systems offering surgeons advancements in precision and visualisation whilst potentially mitigating the limitations of surgical ergonomics faced by manual LS [3–5]). There is a debate regarding the extent to which RALS has improved minimally invasive surgery compared to LS. In particular, the area of musculoskeletal demand is an active field of research with a recent review by Shugaba *et al.* [3] aiming to answer whether all minimally invasive surgery should be robotic- this scoping review takes a distinct approach.

Instead of focusing solely on comparative outcomes of LS and RALS, this review aims to systematically map the current available literature that uses electromyography (EMG) to evaluate muscular activation of surgeons during these procedures collectively and individually. This is because EMG provides valuable insight into factors that affect coordination, fatigue, and the surgical biomechanical demands previously described. This scoping review seeks to identify and describe how EMG is being used across the entire field of minimally invasive surgery. For example, which muscle groups are more commonly assessed, methodologies used, the reported patterns and lengths of EMG data collection, and ultimately identify what the current gaps are within the literature. Considering the exploratory nature of this review, which differs from a more focussed approach of a systematic review, a broader research question was utilised to broaden the scope of the search, making a scoping review methodology appropriate [6]. A systematic review with meta‑analysis would require methodologically similar studies and a narrowly focussed question, conditions not yet met within the overall EMG literature.

While a comprehensive review of the effects of EMG during RALS and LS is an essential tool to guide patient care, it remains uncertain as to whether the available literature is sufficient to conduct quantitative pooling of data (i.e., a meta-analysis). Therefore, undertaking a traditional systematic review and meta-analysis, with a tightly focussed research question would be premature [7]. Consequently, we elected to undertake a scoping review. This approach retains the systematic approach to literature searching but aims to map out the current state of the research [7]. Using the JBI scoping review guidelines [8], a scoping review aims to use a broad set of search terms and include a wide range of study designs and methods (in contrast to a systematic review [8]). This approach has the benefit of clarifying key concepts, surveying current data collection approaches, and identifying critical knowledge gaps.

### Objectives

We aimed to provide an overview of existing literature concerning EMG in MIS. Our three specific objectives of this scoping review were to (1) conduct a systematic search of the published literature concerning EMG during LS and RALS, (2) map characteristics and methodologies used across these surgical modalities, and (3) provide recommendations for the advancement of the research area. This review will complement the current work of meta-analyses in this field by offering a unique insight into research concerning the biomechanical aspects of surgical performance, EMG.

### Review questions

*Primary review question:*

- What is the current evidence regarding EMG of surgeons performing LS and RALS settings?

*Secondary Review Questions:*

- Are there any identifiable trends in EMG research over time regarding LS and RALS settings (this aims to characterise the maturity and evolution of the evidence base over the 28-year period)?
- Are different methodologies utilised during EMG studies (e.g., variations in intervention, electrode placement, outcome measures)?

## Methods

The methodology for this scoping review was completed using the Joanna Briggs Institute (JBI) scoping review guidelines [8] and was reported according to the PRISMA-ScR checklist [9] (S1 File). The protocol of the review, outlining the objectives was registered with PROSPERO before searches were made (Study ID: CRD420251023254. Available at: https://www.crd.york.ac.uk/PROSPERO/view/CRD420251023254). This review was registered as a systematic review due to the lack of option to register as a scoping review. Aside from this, the review followed the details of the registered protocol.

### Inclusion/exclusion criteria

As advised for scoping reviews, the inclusion criteria were developed using the Population, Concept and Context (PCC) framework [8]. Studies were included if they investigated any surgical setting involving laparoscopic abdominal surgeons and used EMG to measure electrical activity, ergonomic workload, or muscular stress in any muscle group. Eligible sources encompassed clinical trial protocols, and peer-reviewed research, quantitative, qualitative, or mixed-methods. A dedicated search of grey literature was not performed. This was due to initial pilot searches revealing a high volume of irrelevant records that lacked EMG metrics and did not meet the inclusion criteria of the review. Conference papers and conference abstracts were also not included. Broad inclusion was essential to capture EMG research trends and identify application gaps, particularly given the limited frequency of follow-up studies in this clinical field due to access challenges. Key exclusion criteria consisted of: meta-analyses or literature reviews or studies focusing on non-abdominal/non-minimally invasive surgery. During the screening process, “wrong study design” was defined as any non-empirical or secondary research, such as commentaries, editorials, or conference abstracts without full-text availability. “Wrong outcome” primarily referred to studies where EMG was used for anything other than assessing surgeon workload. This includes measuring patient muscle responses.

### Search strategy

Initially the search was made in Medical Literature Analysis and Retrieval System Online (MEDLINE) and Academic Search Ultimate. The full search strategy was then developed using titles and abstracts to identify key words that would be needed to create an all-encompassing search within the titles and abstracts of the texts. The search strategy was then used and adapted to each database including Web of Science, Scopus, PubMed, MEDLINE, ERIC, EMBASE, AMED and Academic search ultimate. Each individual search line was then peer-reviewed by the chief investigator LH and a research librarian, which was then altered based on feedback. Language restrictions were set to exclude any literature not in English. The final searches were conducted on Web of Science (Clarivate), Scopus (Elsevier), PubMed, MEDLINE (Ovid), ERIC (EBSCO), EMBASE (Ovid), AMED (EBSCO), and Academic Search Ultimate (EBSCO). Sources that were included were hand-searched for other articles that may be eligible in the review. The final searches were conducted in March 2025 and coverage of the search included sources from any date until the search date. To improve transparency and reproducibility, the MEDLINE search strategy was structured by grouping key concepts and combining them using Boolean operators. Specifically, electromyography related terms and laparoscopic or minimally invasive surgery terms were searched as separate concept blocks joined using AND. The full search strategy that includes the search string for each included database can be found in S2 File. The full MEDLINE (Ovid) search strategy was as follows:

(exp

Electromyography/

OR (Electromyograp* OR EMG).ti,ab.

)

AND

(

 exp Laparoscopy/

 OR exp Hand-Assisted Laparoscopy/

 OR exp Laparoscopes/

 OR exp Cholecystectomy, Laparoscopic/

 OR (

  Laparoscop*

  OR endoscop*

  OR (

   (minimally-invasive OR keyhole OR video-assisted OR “video assisted” OR “belly button”)

   adj3 (surg* OR procedure* OR intervention* OR operation*)

   )

  ).ti,ab.

)

The term “robotic” and related terms were not included in the final search strategy following a sensitivity analysis. Specifically, additional searches incorporating robotic-related keywords were conducted; however, these did not identify any additional eligible records beyond those retrieved by the final search strategy. These terms were therefore excluded to avoid redundancy without compromising the completeness of the search.

### Study selection

All selected studies were uploaded to Rayyan (Qatar Computing Research Institute, Doha, Qatar) where they were stored and then duplicates removed initially by Rayyan’s duplication detection algorithm which was used to highlight potential duplicates and then the primary reviewer (MD) manually deleted manuscripts that were correctly identified as duplicates. Articles with the same outcomes such as the preprint and peer-reviewed versions were also consolidated using this process. The titles and abstracts of each article were then screened by the primary reviewer (MD) and then reviewed by a second independent reviewer (LH). Any differences in opinion about source inclusion were resolved in discussion (with CG). Full text screening against the inclusion and exclusion criteria then commenced after uploading the articles to Rayyan with the same independent review process taking place and any conflicts being resolved. Eighteen papers were excluded due to the full texts being unavailable, but an effort was made to locate all full texts with authors being contacted and requests for access being sent. All publications were included after this step.

### Data charting

Studies selected for analysis in this review were charted using a custom form which was created for this review. The custom data extraction form was created in accordance with JBI guidelines [8]. When multiple sources of the same research were found, the research was condensed into one source material to reduce the chances of double counting. Study characteristic data extracted into table format included: Author name, surgical modality, location, design, aims and summary of results. This table was created using guidance from the Template for Intervention Description and Replication (TIDieR) [10] and is shown below. Data are presented in diagrams and tables, supported by text summaries that highlight significance and relevance of each section.

### Critical appraisal of individual sources of evidence

In accordance with the PRISMA-ScR guidelines and the JBI manual for scoping reviews, a formal critical appraisal or risk-of-bias assessment of the included sources was not performed [8]. The rationale was based on the exploratory nature of the objectives of this review, aiming to map methodologies and identify gaps within the field rather than to assess the quality of the research or provide any clinical recommendations. This approach allowed the review to be more inclusive of a wide variety of studies that add to the overall assessment of technical and methodological trends that may otherwise have been omitted in a synthesis focused solely on the quality of research.

## Results

### Study characteristics

**Fig 1:**
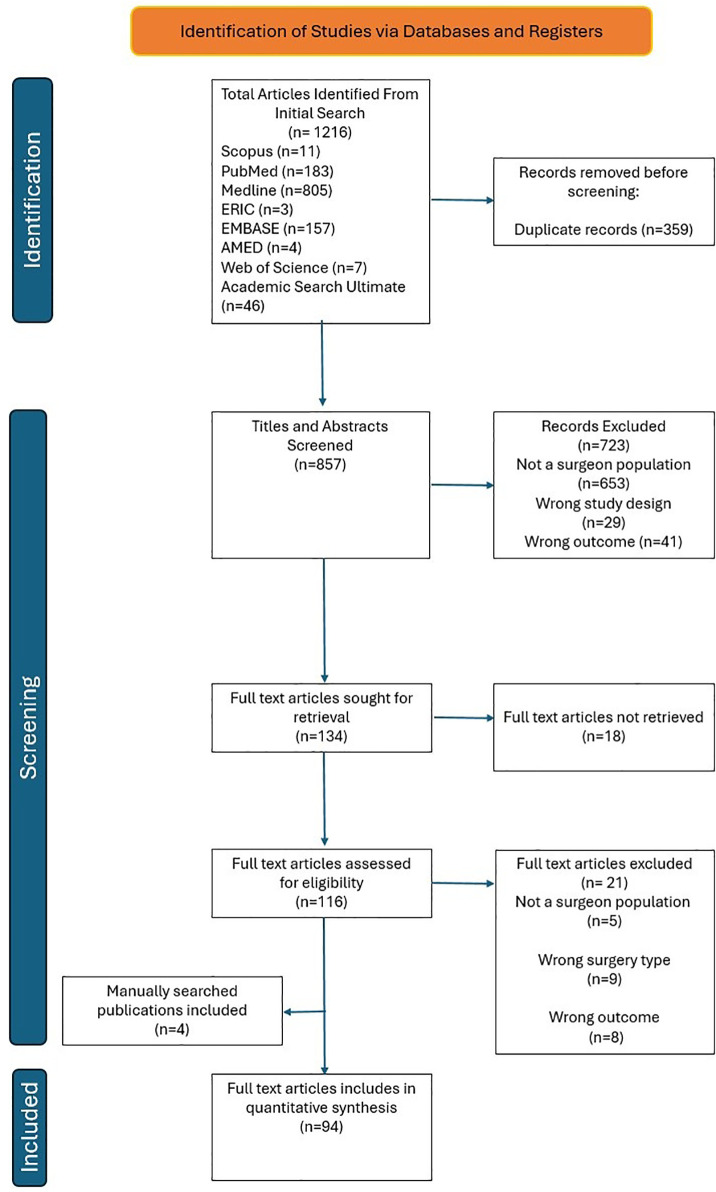
PRISMA-ScR flow diagram of included studies.

1,216 studies were identified during the initial search. Following deduplication in Rayyan, 857 titles and abstracts were screened against the inclusion criteria. When testing for eligibility, a total of 116 studies were assessed; 94 unique studies were included [11–102]. This is demonstrated below in Fig 1.

The analysed 94 studies were categorised as being conducted in a manual LS (n = 62) setting, standalone RALS setting (n = 5), comparative RALS vs LS setting (n = 12) or isolated laparoscopic tasks setting (n = 15). Full descriptions of each study are shown in Table 1 and distribution of task are shown in Table 2.

**Table 1:** Included study characteristics. Where raw data was not available for percentage calculation, *p* value was reported. If results showed no significance, this was stated and the raw data was not presented.

Author Name	Surgical Modality	Location	Design	N of Participants	Aims	Results
Al-Qaisi et al. (2024)	Laparoscopic surgery	Department of Surgery at the American University of Beirut - Medical Centre.	Randomised cross-over study	20 participants	To compare work postures of seated and standing surgeons on physical and subjective measures during simulated laparoscopic tasks.	The difficult task was associated with a significantly higher electromyography activity in the upper trapezius muscle (6.7%MVC vs 7.6%MVC) (p < 0.041) compared to the easy task when sitting.
Alandi- Racafull *et al.* (2023)	Laparoscopic surgery	Universitat Politècnica de València, Spain.	Randomised control trial	17 participants	To assess muscle activity and fatigue of surgeons using two laparoscopic tools.	The prototype tool indicated a lower tendency towards muscle fatigue when comparing the groups for the biceps (23.7%), deltoid (2.7%), and trapezius (3.5%) muscles, while the control tool showed lower fatigue for the flexor carpi radialis muscle (7.4%).
Alhusuny *et al.* (2022)	Laparoscopic surgery	The University of Queensland, Australia	Cross-sectional study	17 participants	To examine changes in muscle fatigue of neck and shoulder muscles of visually impaired surgeons during simulated surgical tasks in two-dimensional and three-dimensional viewing modes.	A downward shift in the median frequency of muscle activity (-15.15%), indicating increased fatigue in the cervical erector spinae and anterior deltoid muscles during more complex tasks.
Alleblas *et al.* (2015)	Laparoscopic surgery	Radboud University Medical Center in Nijmegen, Netherlands.	Randomised control trial	10 participants	To compare the physical workload and muscle activity of surgeons between conventional laparoscopic surgery and single-incision laparoscopic surgery.	Data from 14 out of 16 muscles indicated an increased physical workload in single-incision laparoscopic surgery compared to conventional laparoscopic surgery with a difference of 46.5% difference in the musculus longissimus, 232.5% in the trapezius and 167.5% in the deltoids.
Armijo *et al.* (2019)	Laparoscopic surgery and robot-assisted laparoscopic surgery	University of Nebraska Medical Center.	Observational study	30 participants	To determine differences in muscle effort and fatigue of the upper limb of surgeons between laparoscopic and robotic-assisted surgical approaches.	%MVC was significantly different between the two surgical approaches (p < 0.001) for the measured muscle groups, with differences of 12.2% for the trapezius, 2.6% for the deltoids and 3.5% for the forearm muscles.
Armijo *et al.* (2022)	Laparoscopic surgery	Not specified	Prospective study	18 participants	To assess ergonomics between male and female surgeons while performing laparoscopic procedures.	Female surgeons exhibited a higher muscle activation in the upper trapezius (32% vs 23%), flexor carpi radialis (33% vs 16%), and extensor digitorum (13% vs 10%) compared to men, indicating an increased physical workload.
Asadi *et al.* (2021)	Laparoscopic surgery	Unspecified large academic teaching hospital system.	Observational study	12 participants	To assess the physical and cognitive ergonomics of surgeons during robotic and traditional laparoscopic surgeries.	No significant reductions in the median frequency in the laparoscopic group, while the robotic group showed reductions in the upper trapezius and extensor digitorum muscles, ranging from 3-27% reductions.
Athanasiadis *et al.* (2021)	Laparoscopic surgery	Not specified	Observational study	20 participants	To assess the intraoperative workload and ergonomic factors of surgeons during laparoscopic procedures.	Trainees exhibited less electromyography activity (%MVC) in most muscle groups and spent less time in demanding muscle contractions (deltoid -41.09% and trapezius -39.91%) compared to attendings.
Bartolomeo *et al.* (2012)	Laparoscopic surgery	Not specified	Repeated measures study	15 participants	To investigate the effects of induced mental stress on the muscle activation of surgeons during laparoscopic training.	The induced stress situation resulted in a significantly larger activation of the arm (right biceps p = 0.022, left biceps p = 0.017) and shoulder muscles (right shoulder p = 0.006, left shoulder p = 0.028) with specific differences noted in the right and left forearms, Biceps Brachii, and shoulder muscles.
Berguer *et al.* (1997)	Laparoscopic surgery	University of California Davis Medical Center, Veterans Administration Northern California System of Clinics, Martinez, California, USA	Observational study	21 participants	To compare the upper extremity discomfort and muscle contraction of surgeons during laparoscopic and open surgical tasks.	Surgeons reported a significant increase in forearm flexor muscle contraction during laparoscopic tasks with an increase of 55% (p = 0.01) and significant increases in deltoid (p = 0.02) and extensor muscle contractions (p = 0.05) with task repetition.
Berguer *et al.* (1998)	Laparoscopic surgery	University of California Davis and California State University, Sacramento.	Randomised control trial	18 participants	To compare forearm and hand muscle contractions of surgeons during laparoscopic and open instruments under different grasping loads and wrist angles.	Increased muscle contraction, with a linear relationship between peak electromyographic root mean square voltage and muscle force (0.8 < r < 0.98) across all forearm muscles tested.
Berguer *et al.* (1999)	Laparoscopic surgery	University of California Davis, Sacramento, California, USA.	Randomised self-controlled experiment.	18 participants	To compare forearm and thumb muscle tension of surgeons during laparoscopic instruments with different grips.	Higher electromyographic signal with laparoscopic instruments compared to the standard instrument, with the palm grip showing lower muscle tension than the finger grip at 45 degrees for forearm muscles (p *=* 0.001).
Berguer *et al.* (2001)	Laparoscopic surgery	California State University-Sacramento and the annual meeting exhibit hall of the American College of Surgeons.	Repeated measures study	8 participants	To correlate upper extremity working angle with muscular effort and strain of surgeons using laparoscopic instruments.	Working with laparoscopic instruments at a horizontal angle greater than 45 degrees significantly increased the workload of the flexor digitorum superficialis and deltoid muscles (p < 0.001 for horizontal and p < 0.01 for vertical).
Berguer *et al.* (2003)	Laparoscopic tasks	A semienclosed ergonomics station in the exhibit hall at the Annual Meeting of the Society of American Gastrointestinal Endoscopic Surgeons	Non- randomised 2-condition trial	21 participants	To investigate effects of laparoscopic tasks on the muscular demand of surgeons compared to open surgery tasks.	Laparoscopic tasks resulted in higher average EMG amplitudes in all three muscles (thumb, p = 0.02; forearm flexor, p = 0.01; and deltoid, p = 0.01) and higher peak EMG in the thumb (p = 0.04) and deltoid (p = 0.02) muscles compared with open knot-tying.
Berguer *et al.* (2006)	Laparoscopic surgery and robot-assisted laparoscopic surgery	8th World Congress of Endoscopic Surgery, New York, NY.	Randomised control trial.	10 participants	To compare effects of robotic laparoscopic surgery and standard laparoscopic surgery on the performance, error rates, and physical and mental workload of surgeons.	Robotic technique resulted in decreased thumb muscle forces and possibly reduced mental stress compared to the standard laparoscopic technique (-66.67%).
Berquer *et al.* (2002)	Laparoscopic surgery	University of California, Davis School of Medicine, and the Society of American Gastrointestinal Endoscopic Surgeons conference in Atlanta, Georgia	Randomised control trial	21 participants	To determine the optimum operating table height with discomfort and muscular demand of surgeons during laparoscopic surgery.	Deltoid electromyographic values (%MVC) decreased significantly at lower table heights (p < 0.0006) with a difference of –41.18%, and trapezius electromyographic values also showed significant decreases at various heights (p < 0.0001) with a difference of –50%.
Brown- Clerk *et al.* (2011)	Laparoscopic surgery	University of Nebraska-Lincoln, Lincoln, NE, USA, and TNO Work and Employment, Hoofddorp, The Netherlands.	Randomised control trial	26 participants	To determine the optimal ergonomic placement of novel electrosurgical hand controls on the functionality and physical discomfort of surgeons.	Hand control design one resulted in a significantly greater actuation force for three of the four tasks with minimal forearm muscle activation, indicating a reduced muscular demand: **Control Design 1 (CD1)** Task 2 vs. Task 1: 0% (17.5% - 17.5%), Task 3 vs. Task 1: 2.5% (20% - 17.5%), Task 4 vs. Task 1: -2.5% (15% - 17.5%), Task 3 vs. Task 2: 2.5% (20% - 17.5%), Task 4 vs. Task 2: -2.5% (15% - 17.5%), Task 4 vs. Task 3: -5% (15% - 20%) **Control Design 2 (CD 2)** Task 2 vs. Task 1: 5% (25% - 20%), Task 3 vs. Task 1: 0% (20% - 20%), Task 4 vs. Task 1: -5% (15% - 20%), Task 3 vs. Task 2: -5% (20% - 25%), Task 4 vs. Task 2: -10% (15% - 25%), Task 4 vs. Task 3: -5% (15% - 20%) **Control Design 3 (CD 3)** Task 2 vs. Task 1: 5% (27.5% - 22.5%), Task 3 vs. Task 1: 0% (22.5% - 22.5%), Task 4 vs. Task 1: -10% (12.5% - 22.5%), Task 3 vs. Task 2: -5% (22.5% - 27.5%), Task 4 vs. Task 2: -15% (12.5% - 27.5%), Task 4 vs. Task 3: -10% (12.5% - 22.5%).
Buchel *et al.* (2010)	Laparoscopic bladder dissection	The University Hospitals of Trondheim, Norway, and Tübingen, Germany.	Randomised control trial	25 participants	To evaluate effectiveness, efficiency, and satisfaction of different laparoscopic instrument handles on the physical workload and muscle strain of surgeons.	Muscle strain during tasks was significantly different among the instruments, with the Volargrip handle showing a mean of 7 muscle activations compared to 10 for Ethicon and Tyco handles (hands, forearm muscles, trapezius, biceps). The Volagrip showed an average activation of 17%MVC, the Ethicon averaged 13%MVC and the Tyco averaged 13%MVC.
Chandra *et al.* (2014)	Laparoscopic surgery	Indian Institute of Technology Madras, Chennai, India, and INRIA Demar team, Montpellier, France.	Repeated measures study	8 participants	To analyse fatigue-induced hand tremor and its impact on the positioning accuracy of surgeons during laparoscopic surgical manipulation.	Median frequency of surface electromyography signals decreased by more than 20 Hertz throughout the laparoscopic tasks (-18.95%), indicating an increased muscular demand and fatigue in the hand and forearm.
Chandra *et al.* (2017)	Robot-assisted laparoscopic surgery	the Indian Institute of Technology Madras	Repeated measures study	16 participants	To assess muscle fatigue of laparoscopic surgeons during a series of surgical techniques.	Experienced surgeons exhibited a lower muscular demand in later tasks than in earlier techniques with fatigue being reduced by 23.08% compared to less experienced surgeons.
Dalager *et al.* (2019)	Robot-assisted laparoscopic surgery	Odense University Hospital, Denmark; Karolinska University Hospital, Sweden.	Experimental design with a pilot study approach	6 participants	To assess physical workload and muscle load of surgeons during robotic-assisted laparoscopy using different chair types.	High levels of static and mean muscle activity (40–107%), with an increased perceived physical exertion in the neck muscles, erector spinae, and trapezius, indicating a need for improved ergonomics in robotic surgery.
Dalager *et al.* (2020)	Laparoscopic colorectal procedures	Zealand University Hospital in Roskilde and Aarhus University Hospital, Denmark	Paired cross-sectional study	13 participants	To compare muscular workload, work posture, and perceived physical exertion of surgeons when performing laparoscopic and robotic-assisted laparoscopic surgery.	Surgeons performing laparoscopic surgery exhibited a static muscle activity level of ~3% of maximum electromyography, a median level of 6–8%, and a peak level of 10–15% in the trapezius, neck and forearm muscles.
Dalsgaard *et al.* (2020)	Laparoscopic hysterectomy	Department of Gynecology, Aalborg University Hospital, in Aalborg, Denmark	Paired cross-sectional study	12 participants	To study the musculoskeletal workload of experienced surgeons during laparoscopic surgery compared with robotic assisted laparoscope.	RALS is significantly less physically demanding and is perceived to be less strenuous than LS.
Emam *et al.* (2002) (a)	Laparoscopic bowel suturing	Ninewells Hospital and Medical School, University of Dundee, Scotland	Randomised control trial	10 participants	To determine the optimal physical alignment, visual display, and direction on the intracorporeal laparoscopic bowel suturing of surgeons.	Suturing in the vertical plane resulted in a significantly lower muscle work (p < 0.005) and less fatigue compared to horizontal suturing in the forearms, biceps, and deltoids. By muscle the differences in groups were as follows: Forearm flexors –70.91%, forearm extensors –81.94%, biceps –172.58%, triceps –202.45% and deltoids –319.88%
Emam *et al.* (2002) (b)	Laparoscopic suturing	Ninewells Hospital and Medical School, University of Dundee, Scotland.	Clinical control trial	10 participants	To compare the performance parameters and muscle work of the surgeon’s dominant upper limb during different laparoscopic manipulation setups.	Degradation in performance with the o-optical axis manipulation to the dominant hand, showing an increased muscle work and fatigue compared to the other setups. The difference in theta angle between groups ranged from –35%--61.04%.
Ghasemi *et al.* (2023)	Laparoscopic endoscopy	Physical Medicine and Rehabilitation Department, School of Medicine, Iran University of Medical Sciences, Tehran, Iran.	Quasi experimental design	15 participants	To design and evaluate an endoscope holder on the static load and musculoskeletal disorder risk factors of laparoscopic surgeons.	The new endoscope holder significantly decreased the average electrical activity in the lateral deltoid (-30.45%) and biceps brachii muscles (-31.37%), indicating a reduced muscular demand during endoscopy.
Gonzalez *et al.* (2020)	Laparoscopic surgery	Centro de Cirugía de Mínima Invasión Jesús Usón, Badajoz, Spain.	Randomised control trial	10 participants	To assess a novel design of the laparoscopic gripper handle and compare its ergonomic features on the physical state of surgeons with a commonly used instrument.	The novel design showed a lower activity in the extensor carpi ulnaris and extensor digitorum muscles, indicating a reduced muscle stress during tasks with reductions in muscle activation observed (%MVC) when comparing the groups in the extensor digitorum (-19.75%) and brachioradialis (-16.13%).
Gonzalez- Sanchez *et al.* (2017)	Laparoscopic surgery and robot-assisted laparoscopic surgery	Two hospital sin Malaga, Spain	Cross-sectional study	1 participant	To analyse the fatigue experienced by surgeons on their muscle function, self-perceived fatigue, and postural balance during and after robotic and laparoscopic surgery.	The chief surgeon in the robotic protocol experienced a significant increase in fatigue, with muscle activation frequency slope values showing a decrease of 6.45 Hz/min in the trapezius muscle compared to the assistant surgeon, but only one muscle exceeded the threshold of muscle activation that was set to cause acute fatigue. The erector spinae reached 27.33%MVC.
Huang *et al.* (2014)	Laparoscopic surgery tasks	University of Nebraska Medical Center in Omaha, NE, and the University of Nebraska-Lincoln in Lincoln, NE	Cross-sectional comparative design.	9 participants	To compare the physical and mental workloads of surgeons when performing virtual laparoscopic surgical training tasks using a multi-degree of freedom joystick, a commercial manipulator or a training box	There was a similar muscle effort and fatigue of the upper extremity among the three training environments. Subjects with medical backgrounds used a significantly higher muscle effort when they performed the training task using the joystick than the manipulator but used a similar muscle effort between the joystick and the training box (p < 0.05).
Hubert *et al.* (2013)	Laparoscopic surgery and robot-assisted laparoscopic surgery	School of Surgery laboratory, Faculty of Medicine, Nancy, France; INRS (French National Institute for Research and Safety).	Repeated measures study	11 participants	To assess the physical stress and strain of robot-assisted and standard laparoscopic techniques on surgeons during real operations.	Physical workload was significantly greater in standard laparoscopic surgery with a mean score of 5.7 compared to robot-assisted laparoscopic surgery (p < 0.05). Muscle activation was 32.34% higher than RALS in the right trapezius and 70.89% in the left trapezius.
Judkins *et al.* (2006)	Robot-assisted laparoscopic surgery	University of Nebraska Medical Center (UNMC)	Randomised control trial	15 participants	To determine frequency response of EMG signals on the physical state of specific arm and hand muscles during training with the da Vinci Surgical System (dVSS).	Increased median frequency post-training with an 11.13% difference in median frequency in the flexor carpi radialis, 7.26% difference in the, extensor digitorum, and an 11.65% difference in the triceps brachii.
Judkins *et al.* (2009)	Robot-assisted laparoscopic surgery	Not specified	Randomised control trial	20 participants	To investigate the physiological demands of robotic surgery on the physical state of surgeons and assess if training can reduce these demands.	Surgical training decreased muscle work, indicated by a reduction in mean electromyography by 20% and a decrease in electromyography envelopes.
Kawahira *et al.* (2021)	Laparoscopic inguinal hernia repair and high anterior resection.	Jichi Medical University, Japan.	Repeated measures pilot study	3 participants	To evaluate the physical stress on surgeons during laparoscopic surgery and assess the effectiveness of a wearable device in reducing this stress.	The device led to a tendency of decreased electromyography activity in the left iliopsoas muscle (p = 0.055) with minimal change in the right muscle (p = 0.406). This study reported reductions between groups in device-control-ratios of –40% and –24.6% for the left and right iliopsoas muscles.
Keshavarz *et al.* (2016)	Laparoscopic surgery	Southern Illinois University	Observational study	8 participants	To detect objective manifestations of muscle fatigue from electromyography data and estimate the time-to-fatigue of muscles during laparoscopic surgery.	Muscle fatigue was detected with a significant workload, showing a time-to-fatigue of approximately 47.5 to 57.5 minutes for the trapezius and deltoids respectively during the procedures. There was no sign of fatigue in the biceps and triceps muscles.
Khan *et al.* (2020)	Laparoscopic tasks	Centro de Cirugía de Mínima Invasión Jesús Usón in Cáceres, Spain	Observational, cross-sectional experimental study	30 participants	To analyse the muscle activity in back and forearm muscles and assess the influence of surgical experience on the physical state of surgeons during laparoscopic dissection and suturing.	Expert surgeons showed a significantly lower muscle activity compared to novices: trapezius muscle: 16.32% vs. 52.94% of maximum voluntary contraction, forearm flexors: 5.87% vs. 22.20%, forearm extensors: 12.41% vs. 24.20%, and that muscle activity was higher during suturing than dissection.
Koca *et al.* (2015)	SILS and multi-port laparoscopic surgery	Suleyman Demirel University in Isparta, Turkey	Comparative study	12 participants	To evaluate the mental workload and fatigue in fingers, hand, arm, and shoulder on the physical state of surgeons during SILS and multiport laparoscopy.	Time to complete laparoscopic tasks was longer in the SILS group than the multiport surgery group, with a significant decrease in strength and median frequency slope, indicating a higher muscular demand. These differences between the groups were seen in the non-dominant bicep (-0.07%/second), the dominant deltoid muscle (-0.1%/second) and in the non-dominant deltoid (-0.08%/second).
Kong *et al.* (2010)	Laparoscopic surgery	Seoul National University College of Medicine.	Crossover study	27 participants	To analyse the effect of a three-dimensional system on the laparoscopic performance and muscle tension of surgeons compared to a two-dimensional system.	The three-dimensional system significantly reduced the muscular demand, with root mean square values of electromyography activity (%MVC) showing lower levels compared to the two-dimensional system in the expert surgeon group but not the expert surgeon group. In the novice surgeon group, there was a 21.43% increase in muscle activation in the forearm muscles compared to the two D system but in the expert surgeon group there was a 14.29% decrease compared to the two D system.
Kraemer *et al.* (2018)	Laparoscopic hysterectomy	Not specified	Observational study	11 participants	To investigate the effect of a rotatable handle piece on the muscular stress, fatigue, usability, wrist posture, and working precision of surgeons during laparoscopic procedures.	No differences in muscle stress and fatigue between the handle piece conditions, with muscle activity measured via surface electromyography showing no significant variation.
Kramer *et al.* (2023)	Robot-assisted laparoscopic surgery and laparoscopic surgery	Not specified	Explorative comparison study.	5 participants	To investigate the ergonomic aspects of robotic-assisted laparoscopic surgery versus conventional laparoscopic surgery in terms of muscular and cardiovascular demands, posture, perceived workload, and discomfort.	Significant differences in median muscle activity (MVE), static muscle activity, and muscular rest time between robotic-assisted laparoscopic surgery and conventional laparoscopic surgery. The left trapezius (0.22%), right trapezius (6.3%MVE), right extensor digitorum (1.91%), left flexor carpi radialis (1.88%) and right flexor carpi radialis (2.41%) all showed a higher MVE in laparoscopic surgery compared to RALS but the left extensor digitorum (-2.75%) showed decreased levels compared to RALS.
Lee *et al.* (2011)	Natural Orifice Transluminal Endoscopic Surgery (NOTES) and traditional laparoscopic surgery.	Surgical Ergonomics and Human Factors Laboratory at the Maryland Advanced Simulation, Training, Research, and Innovation Center, University of Maryland, School of Medicine.	Randomised control trial	14 participants	To investigate and compare the physical workloads experienced by MIS surgeons when performing NOTES and laparoscopic techniques.	Performing NOTES required eight to nine times higher muscular workload, with a normalised cumulative muscular workload of 1315.8%MVC compared to 153.9%MVC for traditional laparoscopy, a 754.97% difference between the groups.
Lee *et al.* (2014)	Laparoscopic surgery and robot-assisted laparoscopic surgery	Johns Hopkins University School of Medicine and the University of Maryland School of Medicine.	Repeated measures experimental design.	13 participants	To investigate the differences in physical and cognitive workloads and assess their relationship with surgeons’ skill levels between robotic and laparoscopic surgeries.	Cumulative muscular workload from the biceps and the flexor carpi ulnaris with robotic surgery was significantly lower than with laparoscopic surgery (p < 0.05) with muscular workload in the biceps being 29.69% and the trapezius being 54.19% lower in the RALS group compared to the LS group.
Liang *et al.* (2019)	Laparoscopic surgery	University Hospital, USA.	Prospective cohort study.	24 participants	To quantify the ergonomic impact of patient body mass index on ergonomics of surgeons during laparoscopic surgery.	No difference in the average muscle activation of all eight muscle groups and NASA Task Load Index scores during laparoscopic surgery for surgeons operating on patients with a body mass index greater than or equal to 30 compared to those with a body mass index less than 30 (p > 0.05).
Lim *et al.* (2021)	Laparoscopic gastrectomy	The National Cancer Center, 323 Ilsan-ro, Ilsandong-gu, Goyang-si, Gyeonggi-do, Republic of Korea.	Controlled experimental design	3 participants	To evaluate the ergonomic benefits of using augmented reality in laparoscopic surgery.	Laparoscopic surgeon exhibited a substantial decrease in mental demand by 21.1% and physical demand by 12.5% when using augmented reality compared to conventional monitoring.
Lin *et al.* (2007)	Laparoscopic surgery	Baystate Medical Center	Prospective observational study	12 participants	To define the performance and ergonomic characteristics of freedom of movement display use of expert laparoscopic surgeons and provide valid performance measurement data.	No participant exceeded the fatigue threshold of 10% to 14% for long-lasting intermittent work; one participant’s trapezius values were within the threshold range, indicating normal muscular physiology rather than fatigue.
Maithel *et al.* (2005)	Laparoscopic surgery	Beth Israel Deaconess Medical Center.	Randomised control trial	30 participants	To determine if a head-mounted display improves the task performance and reduces muscle fatigue of surgeons during laparoscopic tasks.	Higher electromyographic amplitude indicated an increased muscle activation of 10.79% when comparing video mounted display to the head mounted display group.
Malisetty *et al.* (2024)	Laparoscopic surgery	Unspecified University campus	Experimental study.	18 participants	To assess the muscle activity and fatigue during laparoscopic surgical tasks and evaluate the impact of subjective workload on the performance of surgeons.	Significant differences in muscle activation patterns, with a notable increase in muscle demand observed in the non-dominant hand across the different tasks.
Manansaykorn *et al.* (2009)	Laparoscopic surgery	No specific location stated	Randomised control trial	20 participants	To investigate the influence of working surface height on the task performance and muscle workload of surgeons in hand-assisted laparoscopic surgery.	Muscle workload in the biceps, triceps, deltoids, forearms, and erector spinae varied significantly with table height when table height differed from –15 cm- + 15cm in increments of 5 cm. Difference when comparing table height ranged from -16.76%-263.63% when comparing to the baseline height of 0 cm. Specific EMG data indicated an increased workload at heights above elbow level.
Manukyan *et al.* (2008)	Laparoscopic bowel suturing	Imperial College of Science Technology and Medicine, St Mary’s Hospital, London, UK; Chulalongkorn University, Bangkok, Thailand.	Repeated measures study	20 participants	To investigate the impact of manipulation angles and instrument length on the task performance and muscle workload of surgeons in hand-assisted laparoscopic surgery.	Short instruments resulted in less muscle activity with a mean electromyography value of 29.27 mV s compared to 29.33 mV s for standard-length instruments (2.04% decrease when comparing short instruments to standard length instruments).
Matern *et al.* (2002)	Laparoscopic surgery	University Hospital Freiburg, Germany	Control trial	12 participants	To evaluate the effect of elbow angles on the electromyographic activity required to manipulate different types of MIS handles.	Virtually no significant difference in electromyographic activity between the two elbow angles for any of the five forearm muscles during manipulation of the handles, indicating similar muscle forces required for both positions.
Matern *et al.* (2004)	Laparoscopic surgery	Not specified	Randomised control trial	10 participants	To investigate the muscle strain of surgeons during various dynamic tasks with different instrument handles in MIS.	Axial handle required significantly more muscle activity (approximately 60% RMSmax) compared to other handles, which had muscle activities between 20% and 35% RMSmax during tasks.
Matern *et al.* (2005)	Laparoscopic suturing in gynecology, urology, and visceral surgery.	University Hospital Freiburg, Germany.	Randomised crossover trial	18 participants	To investigate the impact of different monitor positions on the task performance and muscle strain of surgeons during laparoscopic surgery.	Monitor position at eye level required less electromyographic activity in the neck muscles, indicating a reduced muscle strain (%MVC) compared to other positions. There was a 19.05% reduction in muscle activation in Position A compared to Position B, a 28.57% reduction in Position A compared top position C and an 8% reduction in position B compared to position C.
Monfared *et al.* (2022)	Laparoscopic surgery and robot-assisted laparoscopic surgery	The study was conducted at multiple centers.	Prospective comparative study.	20 participants.	To compare the ergonomic risks to surgeons and surgical trainees performing robotic and laparoscopic procedures.	Higher activation levels of the biceps, triceps, and deltoid muscles after laparoscopic surgery compared to robotic surgery (p < 0.05).
Nakajima *et al.* (2017)	Laparoscopic simulation	Chiba University, Japan	Randomised control trial	6 participants	To assess the function of the Surgical Assist Suit and its impact on reducing physical burden on surgeons during laparoscopic surgery.	No significant differences in shoulder stress measurements between the control group and the Surgical Assist Suit group, p *=* 0.1621 and 0.5730.
Nieboer *et al.* (2013)	Laparoscopic surgery	Radboud University Nijmegen Medical Center.	Randomised control trial	24 participants	To evaluate the effect of training the nondominant upper extremity on the physical strain of surgeons during laparoscopic tasks.	Training did not lead to a significant reduction in physical strain on shoulder and upper-arm muscle activity compared to controls, measured by electromyography.
Nishimoto *et al.* (2019)	Laparoscopic resections of colorectal cancer	Chiba University and National Cancer Center Hospital East, Japan.	Crossover control trial	5 participants	To evaluate the effectiveness of a surgical knee rest in reducing the physical stress on surgeons during laparoscopic surgery.	The mean percentage of maximum gastrocnemius muscle effort was significantly decreased in the left gastrocnemius muscle when the surgical knee rest was used, p = 0.027, for the full 100-minute test period and in both gastrocnemius muscles during the first 50 minutes of the test period (Left Gastrocnemius p = 0.008, right gastrocnemius p = 0.046).
Niu *et al.* (2020)	Laparoscopic surgery and robot-assisted laparoscopic surgery	Tianjin University, Key Laboratory of Mechanism Theory and Equipment Design, Tianjin, China; Rocket Force Characteristic Medical Center of PLA, Beijing, China; Medical College of Soochow University, Suzhou, China.	Repeated measures study	7 participants	To evaluate the biomechanical stress of surgeons performing robotic surgery and traditional laparoscopic surgery.	Muscle activation was 57.03% higher in the left flexor carpi radialis, 37.97% in the right flexor carpi radialis, 46.74% in the left brachioradialis, 8.87% in the right brachioradialis, 11.13% in the left biceps brachii 0.10% in the right biceps brachii during robot-assisted laparoscopic surgery compared to traditional laparoscopic surgery but a 19.13% reduction in left deltoid and 32.22% reduction in right deltoid activation during robot-assisted laparoscopic surgery. There was no significant difference in muscle fatigue (p > 0.05) compared to traditional laparoscopic surgery.
Nowakowski *et al.* (2018)	Laparoscopic tasks	Jagiellonian University Medical College, Krakow, Poland.	Quasi-experimental study	10 participants	To evaluate the changes in surface electromyography and associate them to the skill acquisition of surgeons during laparoscopic training	Average number of knots tied increased from 1.7 to 14, with significant changes in muscle activation patterns observed post-training (%MVC). When comparing before and after training, an increase in muscle activation was observed in the dominant side thenar (0.41%), the dominant side deltoids (7.71%), the non-dominant side thenar (19.48%) and the non-dominant side forearm (33.51%). Decreases in activation post training were observed in the dominant forearm (0.57%), dominant trapezius (6.46%), the non-dominant deltoid (24.76%) and the non-dominant trapezius (44.61%).
Pace-Bedetti *et al.* (2019)	Laparopscopic surgery	Hospital La Fe, Valencia, Spain.	Control trial	17 participants	To evaluate the muscular activity and fatigue effects of a conventional instrument versus an instrument with Postural Freedom feature during laparoscopic surgery.	Postural Freedom feature reduced muscular activity in the evaluated muscles compared to the conventional tool in the trapezius by 72.00%, the deltoids by 74.50%, biceps brachii by 33.5% and the flexor carpi radialis by 44.5%.
Panahi *et al.* (2020)	Laparoscopic tasks	Southern Illinois University Edwardsville and Washington University in Saint Louis.	Observational study	12 participants	To detect muscle fatigue and the time-to-fatigue in specific muscle groups and assess the effect of fatigue on the surgical performance during laparoscopic tasks.	Muscle fatigue was detected in 12 of the 16 muscles tested using recurrence quantification analysis, with DET% values indicating significant fatigue; performance improved over time despite the muscle fatigue. The difference in determinism % between the first and last 10 minutes of surgery ranged from 0.3%-36.3%.
Perez- Duarte *et al.* (2013)	Laparoscopic dissection and suturing	Centro de Cirugía de Mínima Invasión Jesús Usón.	Comparative study	30 participants	To analyse the muscle activity in back and forearm muscles and determine the influence of surgeons’ experience during laparoscopic surgery.	Muscle activity was lower in the expert group compared to novices, with a higher degree of muscle effort observed during suturing tasks. In the novice surgeon group, the average trapezius activation was 52.94%MVC compared to 16.32%MVC in expert surgeons’. The average activation in the forearm flexor muscles in the novice group was 22.2%MVC compared to 5.87%MVC in the expert group. The average activation in the forearm extensors muscles in the novice group was 24.2%MVC compared to 12.41%MVC in the expert group.
Perez-Duarte *et al.* (2014)	Laparoscopic tasks	Jesús Usón MIS Centre in Cáceres, Spain.	Crossover study	14 participants	To evaluate the ergonomics of surgeons during LESS surgery through the study of muscular activity, wrist angle, and hand movements, and compare it with conventional laparoscopy.	Muscular activity for the trapezius and forearm extensor muscles was 63.11% lower in the trapezius during conventional laparoscopy compared to the Laparoendoscopic single site approach.
Quick *et al.* (2003)	Laparoscopic surgery	University of Kentucky	Pilot study with a convenience sample	4 participants	To explore how surgical tasks and instrument design affect muscle activation time and evaluate the contributions of forearm and shoulder muscles during MIS.	Cable-tying exercise required the greatest muscle activation, with a significant activation noted in all tested muscles, particularly in the upper arm. With the deltoids showing a relative time of activation of 85.57% when using grasper 1, 72.16% when using grasper 2 and 75% when using grasper 3.
Rodrigues- Armijo *et al.* (2020)	Laparoscopic surgery and robot-assisted laparoscopic surgery	SAGES Annual Conference Learning Center and the University of Nebraska Medical Center.	Cross-sectional experimental design.	15 participants	To determine how self-reported and objectively measured fatigue of the upper limb differs between laparoscopic and robotic surgical training environments.	Higher levels of muscle activation (%MVC) in the biceps brachii, triceps brachii, erector spinae, trapezius, and lower hand and deltoids during laparoscopic procedures compared to robotic-assisted laparoscopy, with the upper trapezius and the deltoids showing 19.12% and 13.26% increases in muscle activation respectively.
Rodriguez *et al.* (2023)	Laparoscopic tasks	No specified location	Comparative pilot study	14 participants	To present a new physical laparoscopy simulator and objectively evaluate the laparoscopic skills of surgeons based on muscle activity quantification.	Proximal and distal arm muscles showed significant differences in electromyography amplitude and muscle activity between expert and novice surgeons, with experts demonstrating less muscle activity in the trapezius p = 0.002, deltoids p < 0.001, biceps p < 0.001 and forearms p = 0.003.
Rousek *et al.* (2012)	Laparoscopic surgery	University of Nebraska Medical Center.	Full factorial, Repeated measures study	26 participants	To evaluate the ergonomic design of electrosurgical hand controls on the usability, comfort, and muscular activation of surgeons.	Optimal control design required the least muscular activation, with specific electromyography data indicating a reduced muscle demand compared to other designs. There was a difference when co9mparing design three and all other designs (p < 0.001).
Sancibrian *et al.* (2014)	Laparoscopic cholecystectomy	Not specified	Repeated measures	8 participants	To assess the ergonomic performance of a new surgical handle design and compare it to a typical ring-handle on the physical state of surgeons during laparoscopic surgery.	No differences in the muscular demand data for the flexor digitorum communis, extensor digitorum communis, and trapezius pars descendens muscles among the surgeons.
Sancibrian *et al.* (2020)	Laparoscopic cholecystectomy	Not specified	Randomised crossover trial	8 participants	To assess the ergonomic performance of a new surgical handle design and compare it to a traditional handle on the physical state of surgeons.	No differences in muscular demand for the flexor digitorum communis, extensor digitorum communis, and trapezius pars descendent among the surgeons.
Sevestre *et al.* (2024)	Laparoscopic surgery	Nantes University Hospital, obstetrics and gynecology department	Controlled cross-over laboratory trial	29 participants	To assess the impact of a workday on the surgical performance of surgeons and understand the mechanisms of fatigue.	Significant variations in muscle activation, with a mean power frequency of 8.5 Hz during the fatigue session compared to 10.2 Hz in the control session, indicating a 20% increased fatigue after a full workday.
Shafti *et al.* (2015)	Laparoscopic surgery	The Sherman Education Centre, Guy’s Hospital, London.	Controlled study	25 participants	To assess the learnability and comfort of the STIFF-FLOP manipulator on the physical state of surgeons in a clinical scenario.	An average of 25% less muscle activity when using the STIFF-FLOP manipulator compared to conventional laparoscopic tools.
Shergill *et al.* (2009)	Laparpscopic colonoscopy	Veterans Affairs Medical Center, San Francisco, California	Observational study	3 participants	To evaluate the pinch force and forearm-muscle activity of surgeons during colonoscopy and identify the tasks contributing to overuse injuries.	Mean peak pinch forces of the right thumb during colon insertion were 10.4 N and 10.1 N, exceeding the injury threshold of 10 N, while forearm-muscle activity also peaked during this phase with his 49%MVC and 48%MVC for the right and left colon insertion respectively.
Shergill *et al.* (2021)	Laparoscopic colonoscopy	University of California, San Francisco, including a Veterans Affairs Medical Center and a safety net hospital system.	Cross-sectional study.	12 participants	To quantify the biomechanical exposures of gastroenterologists during colonoscopy and evaluate the risk factors for musculoskeletal disorders.	Peak thumb pinch force exceeded the risk threshold of 10 N, and the peak muscle activity for the extensor carpi radialis and flexor digitorum superificialis exceeded 30%MVC of maximum voluntary contraction in both forearms. There was a 18.7% decrease in EMG amplitude when using the support arm.
Shiang *et al.* (2022)	Flexible endoscopy	Department of Surgery and Division of Gastroenterology at a large academic center.	Observational study	27 participants	To analyse the provider factors influencing ergonomic strain on the physical state of surgeons during live endoscopic procedures.	Endoscopists with small hand sizes exhibited a significantly higher ergonomic strain in the left anterior forearm (42.67% greater) compared to large hand sizes.
Shiang *et al.* (2023)	Laparoscopic colonoscopy	Washington University in St. Louis.	Observational study	20 participants	To quantify the ergonomic strain experienced by physicians during colonoscopy, focusing on patient-specific factors.	Increased muscular strain (%MVC) was noted in the right trapezius (8.54%), right deltoid (11.59%), and right posterior forearm (11.45%) when performing colonoscopies on females compared to males., particularly in cases involving female patients and those with a body mass index less than 25, with significant correlations to perceived physical demand.
Shimomura *et al.* (2016)	Laparoscopic surgery	Chiba University, Japan	Controlled clinical study	10 participants	To evaluate the usability and ergonomic design of endoscopic dissector handles on the physical state of surgeons.	The designated handle reduced the muscle load in the extensor and flexor muscles of the forearm (p < 0.05).
Shugaba *et al.* (2023)	Laparoscopic surgery and robot-assisted laparoscopic surgery	The study was conducted at two National Health Service sites in the United Kingdom	Observational study	13 participants	To determine the muscle and cognitive demands of surgeons on their physical and mental state while performing live laparoscopic and robotic surgeries.	Robotic surgery was associated with a reduction in musculoskeletal demands, particularly showing a lower electromyographic activity (%MVC) in the right deltoid (38.57%), trapezius (32.77%), and left latissimus dorsi (27.82%) muscles during LS compared to RALS.
Soangra *et al.* (2022) (a)	Robot-assisted laparoscopic surgery	University of California, Irvine	Repeated measures study	26 participants	To objectively assess the surgical skill levels of surgeons using electromyography and movement variability analysis during robotic suturing.	Cumulative muscular workload was 17.29% lower in expert surgeons compared to intermediate surgeons, 79.68% lower inexpert surgeons compared to novice surgeons and 13.79% lower in intermediate surgeons compared to novice surgeons when completing robotic suturing.
Soangra *et al.* (2022) (b)	Laparoscopic Surgery	No specified location	Repeated measures study	26 participants	To evaluate the surgical skills of surgeons using wearable sensors and identify optimal sensor placements for skill assessment.	Significant interaction effects among skill level and surgical tasks for total time (p < 0.01) and root mean square (*p* < 0.05).
Steinhilber *et al.* (2016)	Laparoscopic surgery	Cary, North Carolina.	Randomised control trial	57 participants	To investigate the effects of different laparoscopic handle designs on the biomechanical stress and muscle activity of surgeons during laparoscopic exercises.	No differences in biomechanical stress between the two handle types, with a higher working height associated with an increased trapezius and deltoid muscle activity (p = 0.08 & p = 0.012 respectively).
Steinhilber *et al.* (2017)	Laparoscopic surgery	University Hospital Tuebingen in Tuebingen, Germany	Randomised control trial	57 participants	To evaluate the effect of a laparoscopic instrument with a 360-degree rotatable handle piece on biomechanical stress and precision of surgeons in different areas of a simulated operating field.	The rotatable handle piece reduced muscle activity of the biceps brachii by 27% compared to the fixed hand piece.
Suh *et al.* (2016)	Robot-assisted laparoscopic surgery	University of Nebraska Medical Center.	Randomised control study	15 participants	To investigate the effects of distractions on the robot-assisted surgical performance of surgeons using both objective and subjective measures.	Significant distraction effects were observed for electromyography measures, with there being a 14.68% increase in muscle activity when comparing passive and active groups and a 27.52% increase in muscle activity when comparing passive and interactive groups.
Szeto *et al.* (2010)	Laparoscopic surgery	Local universities and a medical center	Observational study	25 participants	To examine the physical exposure of surgeons on their neck-shoulder muscle activity during different surgical approaches.	Significantly higher muscle activities in the cervical erector spinae and upper trapezius muscles during open surgery, with median amplitudes of approximately 38–40%, compared to endovascular and laparoscopic procedures.
Szeto *et al.* (2012)	Laparoscopic colorectal surgery	The University of Southern Denmark, Aarhus University Hospital, Zealand University Hospital, and Herlev Hospital.	Paired cross-sectional study	13 participants	To compare the muscular workload, work posture, and perceived physical exertion on the physical state of surgeons performing laparoscopic and robotic-assisted laparoscopic surgery.	Surgeons performing laparoscopic surgery exhibited a static muscle activity level of approximately 3% of maximum electromyography, a median level of 6–8 percent, and a peak level of 10–15%.
Thurston *et al.* (2022)	Laparoscopic foregut surgery	Oregon Health and Science University.	Observational study	5 participants	To investigate the objective physical workload and explore the effect of surgeons’ experience level on the physical workload and fatigue development of surgeons during live laparoscopic procedures.	Low experience surgeons had higher levels of muscular workload (% reference voluntary contraction) with low experience surgeons experiencing 152.6% higher muscular workload than higher experience surgeons in the left lower back and 324.5% higher in the right lower back.
Tieken *et al.* (2024)	laparoscopic inguinal hernia repair surgeries	University of Nebraska Medical Center.	Repeated measures study	4 participants	To assess the differences in surgeon ergonomics and evaluate the associations between EMG data and RULA scores on the physical state of surgeons during surgery.	RULA scores indicated a moderate risk of musculoskeletal injury for both muscle posturing (MP) and muscle fatigue (MF) in both groups, with significant differences in muscle activation patterns observed. When comparing muscle activation (%MVC) in laparoscopic inguinal hernia and robotic inguinal hernia repairs there was an overall 60.92% increase in activation across 5 muscles during both mesh placement and mesh fixation combined. This was also translated to fatigue (median frequency) where the laparoscopic group was overall 8.25% higher than the robotic group.
Uchal *et al.* (2002)	Laparpscopic suturing	5 hospitals surgery department	Randomised control trial	46 participants	To compare the operative end-product quality, procedure effectiveness, and surgeon forearm workload on the physical state of surgeons between an in-line handle and a pistol-grip handle during laparoscopic suturing tasks.	Median amplitude (RMS) was 6.25% higher when using the pistol-grip handles compared to the in-line handles.
Uhrich *et al.* (2002)	Laparoscopic surgery	Human Performance Laboratory, Barnes-Jewish Hospital, and Department of Surgery, Washington University School of Medicine, St. Louis, MO, USA.	Control pilot study	8 participants	To assess the muscle activity and compare the effects of fatigue, monitor placement, and surgical experience on the physical state of surgeons during simulated laparoscopic surgery.	Electromyography data indicated that muscle activity exceeded recommended threshold limits (2–5%), with significant increases in muscle activity observed in several muscles (p < 0.05).
Valorenzos *et al.* (2025)	Laparoscopic inguinal hernia repair surgery	The University Hospital of Southern Denmark.	Observational study	4 participants	To analyse the ergonomic impact on the physical state of surgeons of robotic versus traditional laparoscopic surgery.	Muscle activity levels were comparable between robotic and traditional laparoscopic techniques, with specific data on physical workload not provided in the summary.
Wang *et al.* (2017)	Laparoscopic sigmoid colectomy	Not specified	Observational study	1 participant	To quantify and compare the ergonomic stress on the physical state of a surgeon during laparoscopic versus open surgical procedures.	Laparoscopic surgery resulted in significant reductions in the mean muscle activation for the left triceps (4.07%MVC open vs. 2.65%MVC laparoscopic, 35%MVC reduction) and left deltoid (2.43%MVC open vs. 1.32%MVC laparoscopic).
Wright *et al.* (2022)	Laparoscopic uteroscopy	Not specified	Exploratory pilot study	3 participants	To assess the effects of different surgeon positions and ureteroscope types on the muscle activation of surgeons during simulated ureteroscopy.	Forearm extensor was the most heavily utilised muscle (%MVC). The trapezius and deltoid muscles were activated 64.52% and 90.48% compared to sitting, whereas the forearm flexors had 62.26% increased activity during standing compared to sitting activation.
Zarate Rodriguez *et al.* (2018)	Laparoscopic surgery and robot-assisted laparoscopic surgery	Not specified	Cross-sectional study	31 participants.	To objectively and subjectively assess ergonomic differences between robotic and traditional laparoscopic surgery platforms.	Elevated muscle activation in traditional laparoscopic surgery compared to robotic surgery, with specific muscle activation data not provided in the text p < 0.05).
Zhang *et al.* (2017)	Laparoscopic Cholocystectomy	Medical College, Huazhong University of Science and Technology, China	Simulation-based experimental study	14 participants	To explore the physical and mental workloads of laparoscopic surgeons during different phases of LC and their correlation.	Significant differences in workload between different laparoscopic phases, differences in muscle activation (%MVC) showing a 2.53% increase in the separation phase compared to the access phase, a 24.05% increase in the dissection phase compared to access phase and a dissection phase compared to the separation phase showing varying levels of physical demands.
Zihni *et al.* (2014) (a)	Laparoscopic and robot-assisted laparoscopic surgery	Johns Hopkins University, Baltimore.	Pilot experimental study	6 participants	To investigate ergonomic differences in muscle activation between laparoscopic and robotic surgical platforms using Fundamentals of Laparoscopic Surgery tasks.	Significant differences in muscle activation (%MVC), with an increased activation of the right bicep (150.28%) and left deltoid (145.33%) muscles during laparoscopic tasks compared to robotic tasks.
Zihni *et al.* (2014) (b)	Laparoscopic surgery and robot-assisted laparoscopic surgery	Barnes-Jewish Hospital.	Observational study	1 participant	To compare the activation of muscle groups during traditional laparoscopic surgery and robot-assisted laparoscopic surgery using surface electromyography.	Muscle activation was higher during traditional laparoscopic surgery compared to robot-assisted laparoscopic surgery. In the suturing task, the right biceps and right deltoids showed 51.9% and 58.3% lower activation respectively in robotic tasks compared to the laparoscopic task. Activation during the peg transfer task in the right trapezius was 118.8% higher in the laparoscopic group compared to the robotic group. Activation in the trapezius during the cutting task was also 176.9% higher in the laparoscopic group compared to the robotic group.
Zihni *et al.* (2016)	Laparoscopic cholecystectomy, ventral hernia repair, splenectomy, and inguinal hernia repair.	Barnes-Jewish Hospital.	Observational study	1 participant	To compare the ergonomic stress of surgeons associated with primary and assistant surgical roles during laparoscopic surgery.	Differences between the primary and assisting surgeons in terms of muscular activation (%MVC). There was a decrease in muscular activation in assisting surgeons compared to the primary surgeons in the right biceps (28.15%) and right triceps (16.08%). Activation was decreased in the primary surgeon role in the left trapezius (31.64%).

%MVC, percentage of maximum voluntary contraction; EMG, electromyography; Hz, hertz; LC, laparoscopic cholecystectomy; LESS, laparoendoscopic single-site surgery; LS, laparoscopic surgery; MF, muscle fatigue; MIS, minimally invasive surgery; MVE, maximum voluntary electrical activity; N, newton; NOTES, Natural Orifice Transluminal Endoscopic Surgery; RALS, robot-assisted laparoscopic surgery; RMS, root mean square; RMSmax, maximum root mean square; RULA, Rapid Upper Limb Assessment; STIFF-FLOP, Stiffness-controllable Flexible and Learnable Manipulator; SILS, single-incision laparoscopic surgery.

**Table 2:** The number of included studies from each research setting from a total of 94.

Setting	n (%)
LS vs RALS	12 (13%)
Isolated tasks or simulations	15 (16%)
LS	62 (66%)
RALS	5 (5%)

LS, laparoscopic surgery; RALS, robot-assisted laparoscopic surgery.

**Fig 2:**
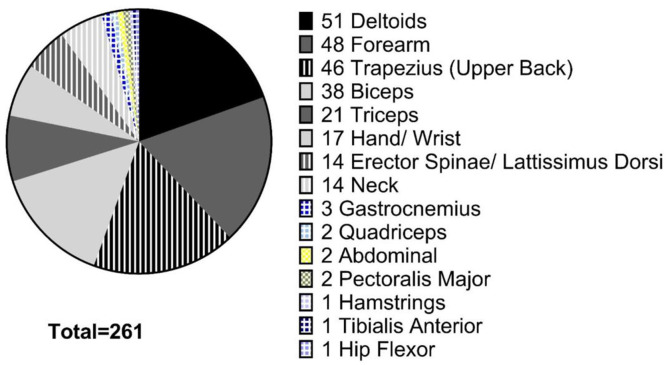
Frequency of muscle groups investigated across all included studies. This figure reflects the distribution of research focus rather than comparative muscle activation between LS and RALS. Modality-specific findings are reported in the Results text.

Of these studies, different muscle groups were investigated in different quantities per study. This means the total tally of muscle groups investigated exceeds the total number of studies: deltoids (n = 51), forearm (n = 48), trapezius (upper back) (n = 46), biceps brachii (n = 38), triceps brachii (n = 21), hand/wrist (n = 17), erector spinae/lower back (n = 14), neck (n = 14), gastrocnemius (n = 3), quadriceps (n = 2), abdominals (n = 2), pectoralis major (n = 2), hamstrings (n = 1), tibialis anterior (n = 1), hip flexors (n = 1). These studies spanned in time of publishing from 1997 until 2025 (up to the search date). The frequency of reported muscle groups is presented in Fig 2. below.

### LS findings

**Fig 3:**
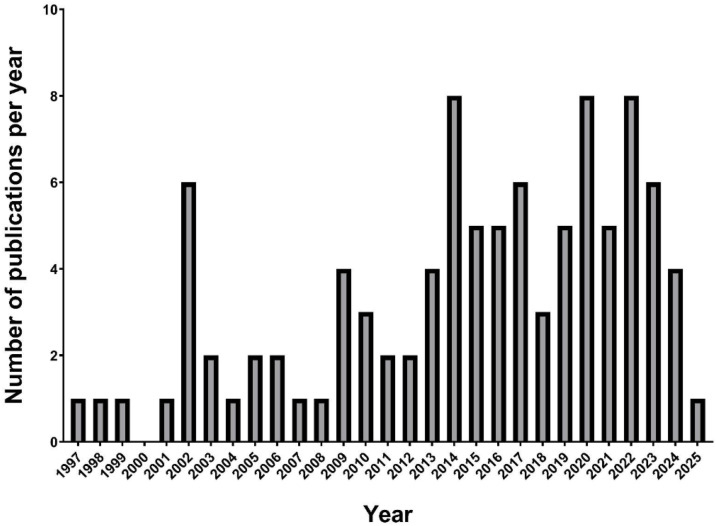
The number of publications by year from 1997 until March 2025.

Studies using EMG during LS contributed the most research papers analysed (n = 62) and were observational than interventional. As shown in Fig 3. below, research since 1997 has been developing an understanding of the ergonomic and physical demands that this type of surgery places on the surgeons. This long-standing investigation was foundational in establishing EMG as a method of quantifying various outcomes of ergonomic demand. For example, research by Berguer *et al*. [19,21,23] used EMG to quantify “physical effort” of surgeons during LS, identifying awkward working angles and body positions which increased muscular demand. This research interest within the field has developed over time, eventually investigating the result of the greater levels of muscular demand (musculoskeletal complaints of surgeons) and using EMG to not just identify the levels of muscular demand, but identify ways in which it can be alleviated in terms of altering technique or introducing new tools [12,14].

### RALS and comparative LS and RALS findings

While EMG in LS has dominated the literature, the growing use of RALS has led to its inclusion in EMG-based studies. The first investigation of EMG during RALS was conducted by Judkins et al. [40]. Since then, only five studies have focussed exclusively on EMG in RALS, as shown in Fig 3. This limited number may reflect a tendency for RALS research to focus on comparisons with LS rather than standalone characterisation. Consequently, more studies (n = 12) have directly compared EMG data between LS and RALS. Research by Chandra et al. [27] and Monfared et al. [61] demonstrated lower EMG activity in RALS, particularly in the trapezius and deltoid muscles. These comparisons are essential to support anecdotal claims that RALS reduces the physical demands placed on surgeons.

### Task-based vs full surgical settings findings

The data also revealed differences between studies that used EMG in simulated or task-based settings versus full surgical procedures. Protocols varied widely, with some studies conducted in laboratory environments and others in clinical settings. This allowed for greater control over variables, such as task duration, complexity, and instrument type, that are difficult to manipulate in observational studies of live surgery, which is the predominant design in LS and RALS research. For example, Berguer et al. [20] compared muscular demand during LS tasks versus open surgery tasks, while Huang et al. [38] examined EMG across different training environments. These studies offer valuable insights into specific aspects of LS, including the impact of instruments, training conditions, and techniques. However, the ecological validity of such findings may be limited, as surgeons rarely perform isolated tasks in clinical practice. Therefore, introducing new tools or techniques into practice requires a larger body of evidence. One such study is Nishimoto et al. [63], which investigated the use of a knee rest to reduce muscular strain during surgery.

### EMG methodology

Although all studies utilised EMG, its role varied depending on the study aims. The following sections outline the most common applications. The most frequently used method for quantifying muscular activation was root mean squared (RMS) amplitude [103]. RMS translates the EMG signal into a measurable amplitude that correlates with the electrical activity generated by motor nerves. It is typically normalised using maximal voluntary contraction (%MVC), which represents the maximum force a muscle can produce when flexed. This allows for comparison of average amplitude over time. For example, Kong et al. [46] used RMS to compare muscle activation between three-dimensional and two-dimensional surgical systems, finding lower RMS values in the former. Similarly, Matern et al. [58] investigated different laparoscopic tool handles and found one design produced significantly higher muscular activity (60% RMSmax) compared to others (25% and 30%).

EMG is also used to assess muscular fatigue, defined as a reduction in the muscle’s mechanical ability to generate force [104,105]. This is commonly measured using median frequency, which decreases over time as fatigue sets in [106]. Studies have used this metric to examine how various factors influence fatigue rates. For instance, Alhusuny et al. [13] observed declining median frequency in the erector spinae and anterior deltoids during complex tasks, while Asadi et al. [16] identified muscle groups more susceptible to fatigue during LS. Both RMS and median frequency provide precise, quantitative insights that move beyond general observations and allow ergonomic effects to be rigorously assessed.

### Scope of EMG application and broader physiological assessment

In addition to its primary use in assessing surgeon ergonomics, EMG has also served as a complementary tool in broader physiological analyses. In these cases, researchers often incorporate additional data collection methods to build a more comprehensive understanding of how specific techniques, tools, surgical procedures, or tasks affect the surgeon. These approaches are outlined below.

A variety of supporting questionnaires were used frequently to assess comorbidities, perceptions of physical and cognitive load, and physical symptoms that may be correlated with EMG. These questionnaires were an important tool in the research of Dalager *et al.* [30] and Shugaba *et al.* [84] where questionnaires were used to gain insight into surgeons’ perceptions about physical wellbeing before quantitative analysis took place. Liang *et al.* [51] used self-report questionnaires in the form of the NASA Task Load Index. This can be used to assess self-perceptions across multiple scales such as temporal stress, physical demand, cognitive demand, performance, effort and frustration [107]. In this study no difference was found in any of the six reported self-perception scales for surgeons when operating on patients with obesity (BMI > 30 kg/m^2^). Questionnaires in research by Kraemer *et al.* [47] were also used to assess the surgeons’ perceptions of tools with rotatable handles vs non-rotatable handles. They reported that there was no preference from the surgeons between the two types of handles. This allowed the objective EMG data to be complimented with context about the surgeons’ perceptions.

A small proportion of the identified literature choose to utilise methods of analysing cognitive demand using objective measures by using an electroencephalogram (EEG) which is a non-invasive method of measuring electrical activity in the brain [108]. Within the research, multiple studies including Asadi *et al.* [16] and Shugaba *et al.* [84] have used EEG as a method of assessing cognitive demands alongside muscular demands using EMG in both LS and RALS. This distinction is critical as it suggests that while RALS may alleviate physical strain, it may simultaneously increase mental workload. The use of EEG alongside EMG is important to contribute to a holistic picture of the effect that RALS has on the surgeon. Just focussing on muscular demand ignores the potential for other factors, such as cognitive demand. This hinders the potential benefits of clinical applications that solve issues within the surgical profession due to reductionist results.

Several studies also complemented the use of EMG with kinematic analysis of surgeons’ limbs. The use of accelerometery, posture and joint angle analysis provided a multi-faceted analysis of the physical demands faced by surgeons. This works in partnership with the use of EMG as it provides external quantification of the biomechanical movements and positions of the surgeons, whilst EMG gives an internal insight on the production of these movements. Within this research, kinematic analysis was most commonly assessed in the upper limbs with varying applications. Chandra *et al.* [27] used accelerometers on the hand and forearm to calculate joint angle of the wrist and elbow when completing repetitive tasks and see whether this influenced wrist tremors. This study found that as the surgeons became more “fatigued” (measured using EMG mean frequency) the tremors at the wrist increased. Perez-Duarte *et al.* [69] also investigated the movement of the right hand and wrist angles using the Cyber Glove motion capture data glove (CyberGlove Systems, San José, CA, USA). This used wrist angle to calculate risk scores comparing LS and single site laparoendoscopy using a modified Rapid Upper Limb Assessment (also used in research by Tieken *et al*. [92]) that assessed risk of injury [109]. It was found that there was a lower risk of injury in single site laparoendoscopy compared to LS. However, the EMG data showed that activity was lower in the trapezius and forearm extensor muscles during LS compared to single site laparoendoscopy. The varied integration of kinematic analyses adds value to quantitative EMG data by providing methodological triangulation through observable meaning within biomechanical landmarks. This added context improves validity of EMG as a tool, strengthening the clinical application of the results by providing interpretation of the surgeons’ biomechanical interaction with the environment.

### Muscle groups assessed

While this research used EMG to investigate the muscular demands of a large proportion of the body, some muscle groups were researched more heavily demonstrated by Fig 2. This section dissects the findings from the muscles that were most frequently investigated: the deltoids, trapezius, forearm, biceps and triceps muscles. The following section synthesises findings across the entire body of included EMG research, irrespective of surgical modality, to describe which muscle groups have been most frequently investigated. Where modality-specific differences between LS and RALS have been identified, these are explicitly stated.

### The deltoids and trapezius muscles

When referring to the deltoid muscles, there are three main muscles: the anterior deltoid, lateral deltoid and posterior deltoid [110]. Similarly, when referring to the trapezius, there are three sections: the upper, middle and lower [111]. Both muscle groups have an important role to play in many functions surrounding the neck and shoulders that surround stability and movement of the joints. In the research, 51 studies chose to investigate the deltoids and 46 chose to investigate the trapezius (and upper back). This is not surprising as within the previous literature surrounding the musculoskeletal discomfort for surgeons, which is a common rationale for research in this field, many studies have pointed to shoulder and neck pain as a serious issue for surgeon wellbeing, time off sick, length of career and even surgical performance [112–114]. Research by Shugaba *et al.*, Hubert *et al.* [39] and Zihni *et al.* [102] all found that there was lower muscular demand in the deltoids and trapezius during RALS compared to LS. In the LS only literature, factors that affect the workload of these muscles with research by Steinhilber *et al.* [87] and Matern *et al* were also investigated [59] higher surgical table height and “awkward” head and neck positions increased activation in the trapezius and deltoids.

### The forearm muscles

The forearm muscles are vastly responsible for fine motor skills required to perform movement and instrument manipulation at the wrist during laparoscopic surgery [115]. Of the 48 studies that investigated the forearm muscles within this field (refer to Fig 3), many studies investigated the effect of differing designs in surgical instruments with varying results. Shimomura *et al.* [83] found that a re-designed, trial dissector handles reduced muscular activation in the forearm compared to a conventional dissector handle. Contrasting research is also prevalent. Sancibrian et al. [75] that found that there was no difference in muscle activation between a “new ergonomic surgical handle” and a traditional surgical tool handle. Although both studies show contrasting results about the effect that surgical tools have on muscle activation, they both act as evidence that the focus of this field is to mitigate hand and forearm strain in surgeons.

Other research that investigated the forearms investigated the muscular demands of completing different tasks with varying complexity. Perez-Duarte et al. [70] found that a higher degree of muscle activation was required to complete tasks such as suturing but Quick *et al.* [71] found that the greatest level of activation in the forearms was during cable-tying exercises. Further investigations showed that using an endoscope produced peak forces that were greater than the recommended levels of activation [79]. Whichever aspect of LS was investigated, the forearm muscles were subjected to high levels of muscle activation during activities that required precise and complex movements, showing that EMG is a trusted method of measuring lower levels of muscle activation caused by fine movements.

### The biceps and triceps muscles

The biceps and triceps muscle groups are responsible for flexion and extension at the elbow joint [116] and play a crucial role in the stability or the arm when manoeuvring instruments into the most optimal position. Research within this field has focussed on the effects that sustained flexion, or extension under load or tension may have on the levels of muscular activation in these muscles. This is supported by research that suggests that the level of activation in these muscles may be lower during RALS than in LS or LS tasks [72,102]. This research utilises EMG to investigate the effects of isometric and isotonic contractions and finds that potentially due to the physical support for the arms during RALS, LS produces a greater level of muscular activation.

## Discussion

Based on the 94 primary studies identified over a 28-year span, EMG is presented as a well-established and reliable tool for quantifying the muscular demands associated with RALS, LS, and LS-related tasks. While consistent findings within the 12 included comparative literature indicate that RALS may result in lower muscular activation than LS, particularly in the deltoid and trapezius muscle groups [39,84,102], these results should be interpreted with caution. It must also be noted that most of the evidence base (n = 62) serves to establish the baseline physical demands of LS. These muscle groups are subject to sustained isometric contraction during LS, a demand that appears to be mitigated by the physical support provided in RALS. Such evidence is important in substantiating anecdotal claims that RALS reduces the physical strain experienced by surgeons [27,61]. This scoping review offers a comprehensive synthesis of EMG research in the context of LS and RALS, an area of growing importance in surgical ergonomics. By systematically mapping 94 studies over a 28-year period, the review identifies key trends, methodological approaches, and gaps in the literature, particularly in relation to muscle activation and fatigue in surgeons. The available comparative studies suggest a trend toward lower muscular activation during RALS than LS, particularly in selected upper-body muscle groups. However, this finding should be interpreted cautiously because the comparative evidence base remains limited, heterogeneous, and was not formally appraised for methodological quality. The significance of EMG in abdominal laparoscopic and robot-assisted laparoscopic surgery lies in the integration with complementary tools such as EEG, kinematic analysis, and questionnaires, providing a multidimensional understanding of surgeon workload. The study’s rigour is demonstrated through adherence to JBI and PRISMA-ScR guidelines, a registered protocol, and a robust search strategy peer-reviewed by experts. This work not only consolidates existing evidence but also lays the groundwork for future meta-analyses and longitudinal studies, with practical implications for surgical training, tool design, and occupational health.

There were clear trends that emerged within this field over time with the first studies emerging in the 1990s and early 2000s being foundational and observational in their nature [19,21,23]. This established EMG as a potentially reliable and rigorous tool to quantify the physical demand that LS subjected on surgeons. With the emergence of RALS, research studies began to take an interest in the comparison between RALS and LS in terms of muscular demand, with studies investigating different aspects of muscular demand including muscle activation with RMS [46,58] and muscle fatigue using median frequency [2,16]. As time has progressed into the 2020s, the number of research papers in this field has increased on average per year, with research looking to optimise and investigate surgeon ergonomics using new technologies. These technologies included wearable sensors, motion capture systems, augmented reality and simulated training [85].

The methodologies involving and surrounding EMG have also varied vastly. Multi-faceted analysis often sees the use of EMG taking both a leading and supporting role within studies. Often EMG has been paired with questionnaire-based assessments to investigate surgeon perceptions of workload [30,51], along with EEG which can objectively provide insight into cognitive demand and act as counterbalance between cognitive and physical demands [16,84]. Kinematic analysis has also been incorporated with EMG research, with accelerometers and motion capture being used to analyse the joint angles and posture of surgeons [27,69,92]. This is important to provide context on the external outputs that muscle activation produces.

The variations in methodology, outcome measures and muscle groups investigated within this literature base shows that EMG is not only trusted, but also a versatile tool in the arsenal of investigating muscular demand. Its ability to be used to detect muscle activation in both fine and gross motor movements, along with isotonic and isometric contractions, makes it, potentially, the ideal tool for the dynamic environment of the operating theatre. That being said, this makes it incredibly difficult to ensure standardised practise between EMG studies as there are many extraneous variables within an operating theatre. Although efforts are made to normalise the data, the unpredictable nature of the surgical environment and the variability within surgeries make this type of research difficult to directly compare. This may offer some explanation as to why there are some contrasting results within this field. An important way to mitigate this risk may be to use other tools such as EEG [16,84], accelerometers [27,69], questionnaires [30,47,84] and motion capture cameras [85].

The impact that these studies could have on the wider clinical setting as well as within teaching and training cannot be understated. It’s role in providing live biofeedback flexibly in a vast range of contexts allows it to move beyond its common research use in the 1990s and 2000s of quantifying muscular demand solely. Research has consistently demonstrated that more experienced surgeons have a reduced muscular demand compared to less experienced surgeons [44,70]. This could mean that a future application for EMG could lie within the training curricula, helping trainee surgeons to objectively track their ergonomic efficiency and develop an awareness about reducing unnecessary muscle activation, hence reducing musculoskeletal injury. EMG may show potential as a training tool, with multiple studies reporting lower muscle activation in experienced surgeons, suggesting a role for biofeedback-informed ergonomic training. Although variables such as procedure type, operative duration, patient characteristics, and intraoperative positioning were not systematically analysed due to heterogeneity and inconsistent reporting, narrative trends indicate that these factors may influence muscular workload. Explicit consideration and reporting of these variables in future studies would enhance translational relevance and applicability to real-world surgical practice and may increase EMG’s use as a training tool.

Furthermore, the review highlights that ergonomic comparison within the surgical setting goes beyond just RALS and LS both individually and when compared. The variation in results when considering different tools highlights a future requirement to consider surgeon ergonomics more carefully when designing tools. Some studies state that the re-designing of instruments can reduce muscular demand in the forearm [83], but others found no difference between the traditional instruments and re-designed instruments [47,75]. This is particularly concerning when some tools were labelled as “ergonomic” handles, proving that there needs to be a closer collaboration between medical engineers and surgeons to make equipment that is both surgically effective and ergonomically efficient.

It is important to note that although this review has highlighted areas of strength in the use of EMG within the literature, it has also highlighted gaps that future research could look to address. Most studies that investigated LS and RALS were cross-sectional or observational studies that offer a brief insight into a surgeon’s muscular demand during a live surgery [16,19,21,23,30,31,61]. However, considering that a lot of these studies use the high rates of musculoskeletal injury among surgeons as a rationale, it is important that an understanding of long-term surgical impact [117]. This means that more longitudinal studies are required to track changes in muscle activation and levels of muscular fatigue throughout a surgeon’s career. In addition, research has begun to investigate how patients’ unique factors affect surgeon ergonomics, such as how BMI was found to influence the muscular demand for a surgeon [51] but has not yet fully explored this. Finally, when considering the comparisons between RALS and LS, there is currently a limited number of studies. This means that with all the muscles in the body, there needs to be more research investigating key areas mentioned to be points of injury such as the shoulders, upper back and wrists [112–114]. When this evidence base builds to more than a few studies on each muscle group, a greater case could be made for the introduction of RALS into more hospital trusts. Addressing the gaps identified within the literature using EMG as a tool will produce a more holistic picture of surgeon ergonomics with greater ecological validity. It is also necessary to acknowledge that there may be the potential for publication bias within this evidence base of identified studies that predominantly reports findings favourable to robotic systems. This may reflect an effect where neutral or negative ergonomic results are not published.

Finally, the number of papers included in this study (n = 94) shows that a future next step may be to conduct a quantitative meta-analysis. This will allow for a quantitative summary to be created of the individual research areas identified within this review, increasing the generalisability of the findings and potentially providing a more objective interpretation of the field.

### Conceptual framework for methodological consistency in EMG-based surgical ergonomics research

The heterogeneity identified across the included studies highlights an opportunity to improve comparability and translational relevance through greater methodological alignment in future EMG-based surgical research. Based on common approaches observed throughout this scoping review, a preliminary conceptual framework is proposed to guide future investigations without imposing prescriptive standards.

### Muscle group selection

The literature demonstrates a strong focus on the deltoids, trapezius, forearm, biceps, and triceps muscles, reflecting their relevance to reported musculoskeletal complaints among surgeons. Future studies may benefit from explicitly justifying muscle selection based on task demands, surgeon posture, and reported injury prevalence, while prioritising these commonly studied upper-limb and shoulder stabilising muscles to facilitate cross-study comparison.

### EMG outcome measures and normalisation

RMS amplitude and median frequency were the most frequently reported outcomes for muscle activation and fatigue, respectively. Consistent use of these metrics, alongside transparent reporting of normalisation procedures (e.g., %MVC), would allow for greater interpretability and allow for synthesis across studies despite unavoidable contextual variation in surgical tasks.

### Task definition and study context

Clear differentiation between simulated, task-based, and full procedural settings is critical, as ecological validity varies substantially across these designs. Future research may benefit from providing detailed descriptions of task duration, complexity, and instrument characteristics to contextualise EMG findings and improve reproducibility.

### Integration of complementary measure

Studies incorporating kinematic analysis, questionnaires, or EEG provided richer interpretation of EMG data by linking muscle activation to posture, cognitive demand, and perceived workload. Future investigations may benefit from adopting multimodal approaches to avoid reductionist interpretation of surgeon workload based solely on muscular demand.

### Limitations

Although this scoping review provides a comprehensive map of the current evidence, there are several limitations that must be acknowledged. Firstly, in accordance with JBI guidelines for scoping reviews, a formal critical appraisal or risk-of-bias assessment was not performed. Consequently, the findings regarding the ergonomic advantages of certain surgical modalities should be viewed as identified trends within the literature base as opposed to evidence used to make clinical recommendation. Secondly, within the literature base, there was a significant imbalance with 62 studies focusing on manual LS and only 12 providing a direct comparison with RALS; this disparity limits the ability to draw broad generalizations regarding robotic ergonomics but does allow for gaps in research and potential trends to be identified. Also, many included studies were small, observational, simulation-based, or involved isolated tasks rather than live operative settings. Therefore, current ergonomic benchmarks may not accurately represent the true musculoskeletal workload experienced by clinicians in the operating theatre.

Furthermore, direct quantitative analysis was made difficult due to the methodological heterogeneity across study methodologies including variations in muscle selection, EMG normalisation techniques and task complexity. Additionally, the focus of this review was on EMG, so other factors such as cognitive or visual strain were not considered, as this was outside the scope of this article. Lastly, no dedicated grey literature search was conducted, and the search was limited to English-language publications only. This may have resulted in the omission of emerging literature.

## Conclusion

This scoping review demonstrates that EMG is a well-established tool for assessing muscular demand in abdominal minimally invasive surgery. While the largest volume of evidence evaluates the ergonomic challenges of LS, a smaller but consistent evidence base suggests that RALS was associated with lower muscular activation, particularly in the upper body. However, this finding should be interpreted cautiously because the comparative evidence base remains limited, heterogeneous, and was not formally appraised for methodological quality. The field has evolved from foundational observational studies to sophisticated, multi-modal investigations incorporating technologies such as EEG, motion capture, and wearable sensors. Despite these advancements, generalisability remains limited due to the predominance of single-centre, cross-sectional designs. Future research should adopt longitudinal approaches to better understand the cumulative impact of surgical practice over a career. EMG may also hold promise as a biofeedback tool in surgical training, helping to reduce musculoskeletal injury through improved ergonomic awareness. Further research is needed to comprehensively quantify surgeon workload during minimally invasive procedures, including the development of a validated surgeon demand index or real-time monitoring tools such as a surgical tachograph, to better predict and prevent musculoskeletal injury. A future meta-analysis may be feasible, but only for carefully defined subgroups with comparable EMG protocols, task types, muscle groups, normalization procedures, and outcome measures.

## Supporting information

S1 File PRISMA-ScR checklist. (DOCX)

S2 File Full search strings for each database. (DOCX)
