# Supplementary material for: Electromyography in abdominal laparoscopic and robot-assisted laparoscopic surgery: A scoping review
Source: PLoS One. 2026 Aug 3;21(8):e0354158. doi: 10.1371/journal.pone.0354158 (PMC13432106; doi:10.1371/journal.pone.0354158)
Supplement: S2 File — (DOCX) [file pone.0354158.s002.docx]

**Medline (Ovid):**

1. Electromyography (EMG)

exp Electromyography/

OR (Electromyograp* OR EMG).ti,ab.

AND

2. Laparoscopic / Minimally Invasive Surgery

exp Laparoscopy/

OR exp Hand-Assisted Laparoscopy/

OR exp Laparoscopes/

OR exp Cholecystectomy, Laparoscopic/

OR (

Laparoscop*

OR endoscop*

OR (

(minimally-invasive OR keyhole OR video-assisted OR "video assisted" OR "belly button")

adj3 (surg* OR procedure* OR intervention* OR operation*)

)

).ti,ab.

**Academic Search Ultimate:**

(

KW DE "ELECTROMYOGRAPHY"

OR (MH "Electromyography")

OR TI (Electromyograp* OR EMG)

OR AB (Electromyograp* OR EMG)

)

AND

(

(

KW (DE "LAPAROSCOPIC" OR DE "LAPAROSCOPY")

OR (MH "Laparoscopy+")

OR (MH "Hand-Assisted Laparoscopy")

OR (MH "Laparoscopes")

OR (MH "Cholecystectomy, Laparoscopic")

)

OR

(

TI (

Laparoscop*

OR endoscop*

OR (

(minimally-invasive OR keyhole OR video-assisted OR "video assisted" OR "belly button")

N3 (surg* OR procedure* OR intervention* OR operation*)

)

)

OR AB (

Laparoscop*

OR endoscop*

OR (

(minimally-invasive OR keyhole OR video-assisted OR "video assisted" OR "belly button")

N3 (surg* OR procedure* OR intervention* OR operation*)

)

)

)

)

**AMED:**

KW ( DE "LAPAROSCOPIC" OR DE "LAPAROSCOPY" OR DE "LAPAROSCOPIC" ) AND TI ( ( (MH "Laparoscopy+") OR (MH "Hand-Assisted Laparoscopy") OR (MH "Laparoscopes") OR (MH "Cholecystectomy, Laparoscopic") ) OR TI ( Laparoscop* OR endoscop* OR ((minimally-invasive OR keyhole OR video-assisted OR "video assisted" OR "belly button") N3 (surg* OR procedure OR intervention OR operation)) ) OR AB ( Laparoscop* OR endoscop* OR ((minimally-invasive OR keyhole OR video-assisted OR "video assisted" OR "belly button") N3 (surg* OR procedure OR ) AND AB ( ( (MH "Laparoscopy+") OR (MH "Hand-Assisted Laparoscopy") OR (MH "Laparoscopes") OR (MH "Cholecystectomy, Laparoscopic") ) OR TI ( Laparoscop* OR endoscop* OR ((minimally-invasive OR keyhole OR video-assisted OR "video assisted" OR "belly button") N3 (surg* OR procedure OR intervention OR operation)) ) OR AB ( Laparoscop* OR endoscop* OR ((minimally-invasive OR keyhole OR video-assisted OR "video assisted" OR "belly button") N3 (surg* OR procedure OR ) AND KW DE "ELECTROMYOGRAPHY" OR TI ( (MH "Electromyography") OR TI ( Electromyograp* OR EMG ) OR AB ( Electromyograp* OR EMG ) ) OR AB ( (MH "Electromyography") OR TI ( Electromyograp* OR EMG ) OR AB ( Electromyograp* OR EMG ) )

**EMBASE:**

(

DE "ELECTROMYOGRAPHY"

OR (MH "Electromyography")

OR TI (Electromyograp* OR EMG)

OR AB (Electromyograp* OR EMG)

)

AND

(

(

DE "LAPAROSCOPIC"

OR DE "LAPAROSCOPY"

OR (MH "Laparoscopy+")

OR (MH "Hand-Assisted Laparoscopy")

OR (MH "Laparoscopes")

OR (MH "Cholecystectomy, Laparoscopic")

)

OR

(

TI (

Laparoscop*

OR endoscop*

OR (

(minimally-invasive OR keyhole OR video-assisted OR "video assisted" OR "belly button")

N3 (surg* OR procedure* OR intervention* OR operation*)

)

)

OR AB (

Laparoscop*

OR endoscop*

OR (

(minimally-invasive OR keyhole OR video-assisted OR "video assisted" OR "belly button")

**PubMed:**

(

"Electromyography"[MeSH Terms]

OR "Electromyography"[Other Terms]

OR electromyography[Title/Abstract]

OR EMG[Title/Abstract]

)

AND

(

(

"laparoscop*"[MeSH Terms]

OR "Hand-Assisted Laparoscopy"[MeSH Terms]

OR "Laparoscopic"[MeSH Terms]

)

OR

(

laparoscop*[Title/Abstract]

OR "hand-assisted laparoscop*"[Title/Abstract]

OR laparoscopy[Title/Abstract]

OR laparoscopic[Title/Abstract]

OR (

(minimally invasive[Title/Abstract]

OR keyhole[Title/Abstract]

OR "video-assisted"[Title/Abstract]

OR "video assisted"[Title/Abstract]

OR "belly button"[Title/Abstract])

AND

(surg*[Title/Abstract]

OR procedure*[Title/Abstract]

OR intervention*[Title/Abstract]

OR operation*[Title/Abstract])

)

)

)

**ERIC:**

(

DE "ELECTROMYOGRAPHY"

OR (MH "Electromyography")

OR TI (Electromyograp* OR EMG)

OR AB (Electromyograp* OR EMG)

)

AND

(

(

DE "LAPAROSCOPIC"

OR DE "LAPAROSCOPY"

OR (MH "Laparoscopy+")

OR (MH "Hand-Assisted Laparoscopy")

OR (MH "Laparoscopes")

OR (MH "Cholecystectomy, Laparoscopic")

)

OR

(

TI (

Laparoscop*

OR endoscop*

OR (

(minimally-invasive OR keyhole OR video-assisted OR "video assisted" OR "belly button")

N3 (surg* OR procedure* OR intervention* OR operation*)

)

)

OR AB (

Laparoscop*

OR endoscop*

OR (

(minimally-invasive OR keyhole OR video-assisted OR "video assisted" OR "belly button")

N3 (surg* OR procedure* OR intervention* OR operation*)

)

)

)

)

``

**Scopus:**

(

TITLE-ABS-KEY(electromyography OR EMG)

)

AND

(

TITLE-ABS-KEY(

laparoscop*

OR "hand-assisted laparoscop*"

OR laparoscopy

OR laparoscopic

OR (

(minimally-invasive OR keyhole OR "video-assisted" OR "video assisted" OR "belly button")

W/3 (surg* OR procedure* OR intervention* OR operation*)

)

)

)

``

**Web of Science:**

TS=(electromyography OR EMG)

AND

TS=(

laparoscop*

OR "hand-assisted laparoscop*"

OR laparoscopy

OR laparoscopic

OR (

(minimally-invasive OR keyhole OR "video-assisted" OR "video assisted" OR "belly button")

NEAR/3 (surg* OR procedure* OR intervention* OR operation*)

)

)
